# Hairy Polyp Causing Infant Respiratory Distress: A Case Report

**DOI:** 10.7759/cureus.114484

**Published:** 2026-08-13

**Authors:** Yousef Ibrahim, Joseph Griffin, David Marriott, Theodoros Valsamakis

**Affiliations:** 1 Ear, Nose and Throat (ENT), Leicester Royal Infirmary, Leicester, GBR; 2 Anaesthetics, Leicester Royal Infirmary, Leicester, GBR

**Keywords:** airway management, congenital, dermoid cyst, embryology, stridor

## Abstract

We present the case of a newborn who presented in respiratory distress with a hairy polyp that was larger than 3 cm arising from the oropharynx. Whilst performing emergency intubation, an oropharyngeal mass was detected causing airway obstruction in association with a cleft palate. The characteristics and extent of the lesion were confirmed through imaging. Surgical removal was undertaken using coblation. Histology confirmed a 34 x 22 x 16 mm lesion consisting of keratinized stratified squamous epithelium with pilosebaceous units and a well-formed tooth bell seen within the specimen. The child made an uneventful recovery and has had complete resolution of symptoms.

## Introduction

Respiratory distress with airway obstruction in the newborn is a life-threatening emergency requiring expertise from a number of experienced specialists [1]. The differential diagnosis of respiratory distress is vast, including simple foreign body, choanal atresia and congenital head and neck malformations such as teratomas, lymphatic malformations, haemangiomas, gliomas and encephalocoeles [2-6].

Hairy polyps have been described in the literature as a cause of neonatal respiratory distress [7]. They may arise from various anatomical subsites, most commonly the lateral nasopharyngeal wall [7]. In a recent review of such lesions, when found in other areas such as the oropharynx, patients usually present at an older age with other symptoms such as feeding difficulties [7]. We present the case of a newborn who presented in respiratory distress with a hairy polyp that was larger than 3 cm arising from the oropharynx.

## Case presentation

A female child was born at 39+5 weeks via elective caesarean section due to foetal abnormalities detected on an antenatal scan indicating ventriculomegaly and absent corpus callosum. The child struggled to oxygenate and was found to be in respiratory distress shortly after birth, necessitating emergency intubation. Whilst performing intubation, an oropharyngeal mass was detected causing airway obstruction in association with a cleft palate. The child was fed through a nasogastric tube whilst obtaining the necessary radiological investigations to plan surgical removal of this mass.

**Figure 1:**
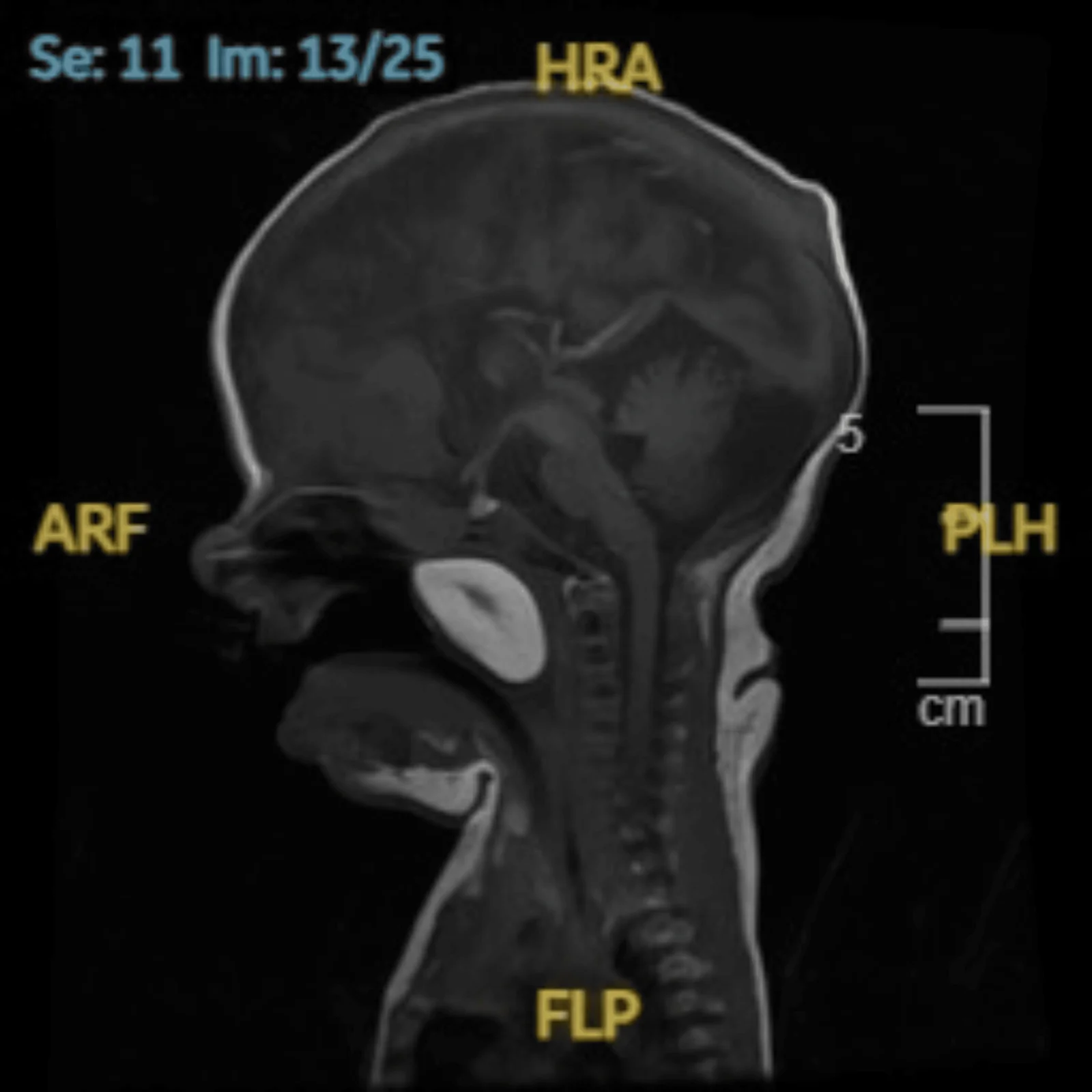
T1-weighted MRI scan showing hairy polyp (sagittal view) MRI: magnetic resonance imaging

**Figure 2:**
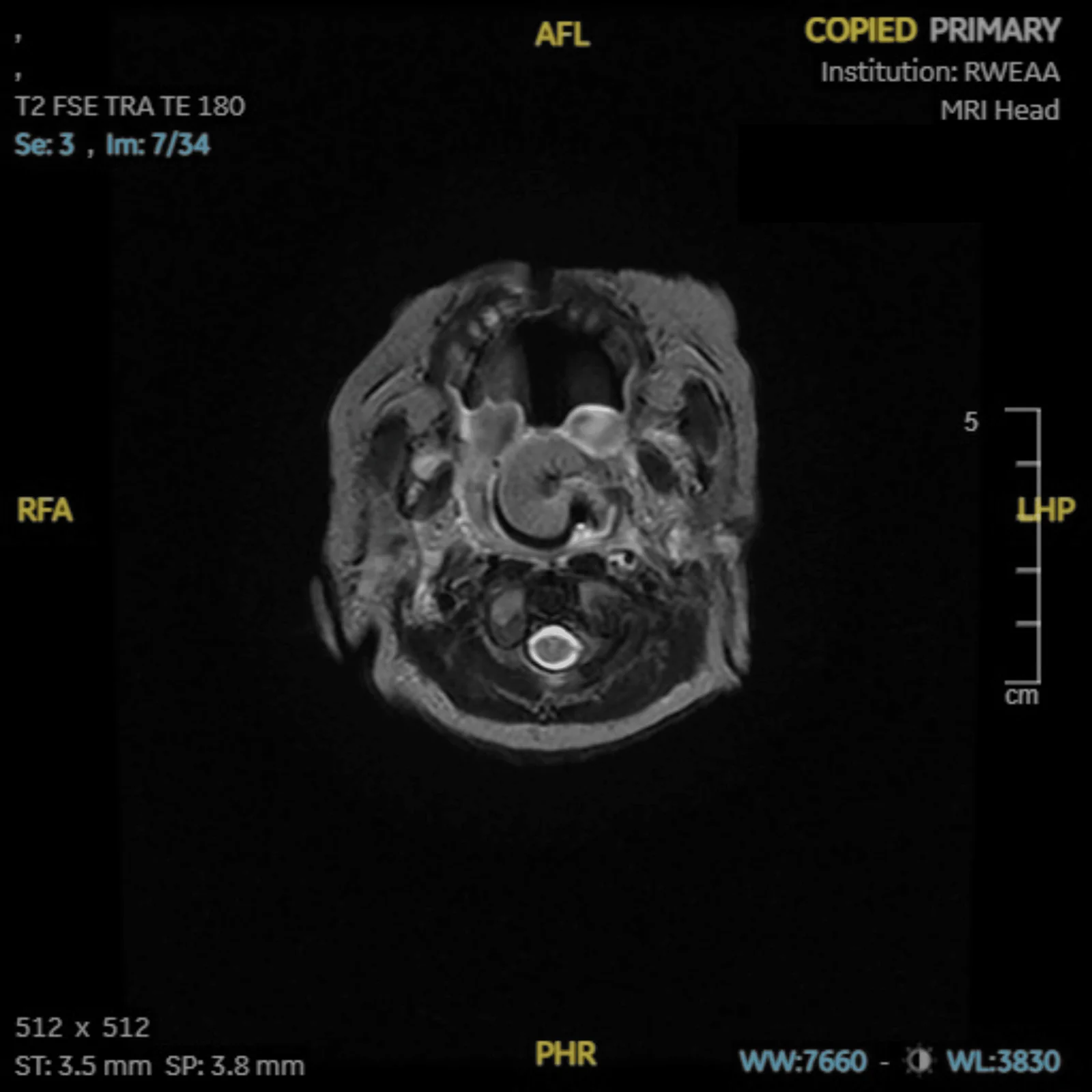
T2-weighted MRI scan showing hairy polyp (axial view) MRI: magnetic resonance imaging

Magnetic resonance imaging (MRI) was performed showing a well-defined polypoidal structure within the oropharynx, measuring 2 x 2.1 x 3 cm. It demonstrated predominantly fatty signal within (high T1, low STIR and isointense T2 signals) with central T1/T2 hypointensity and a focal 'tooth'-like structure at the left lateral aspect of the lesion. There was no pathological diffusion restriction. It lay posterior to the soft palate, extending into the nasopharynx, and was closely related to the left torus tubarius (Figure 1 and Figure 2). Agenesis of the corpus callosum was noted without any other intracranial abnormalities. Following the MRI, a computed tomography (CT) angiogram was performed to ensure this lesion did not have a vascular component to it. This confirmed no aberrant arterial vessels or large-calibre feeding vessels.

**Figure 3:**
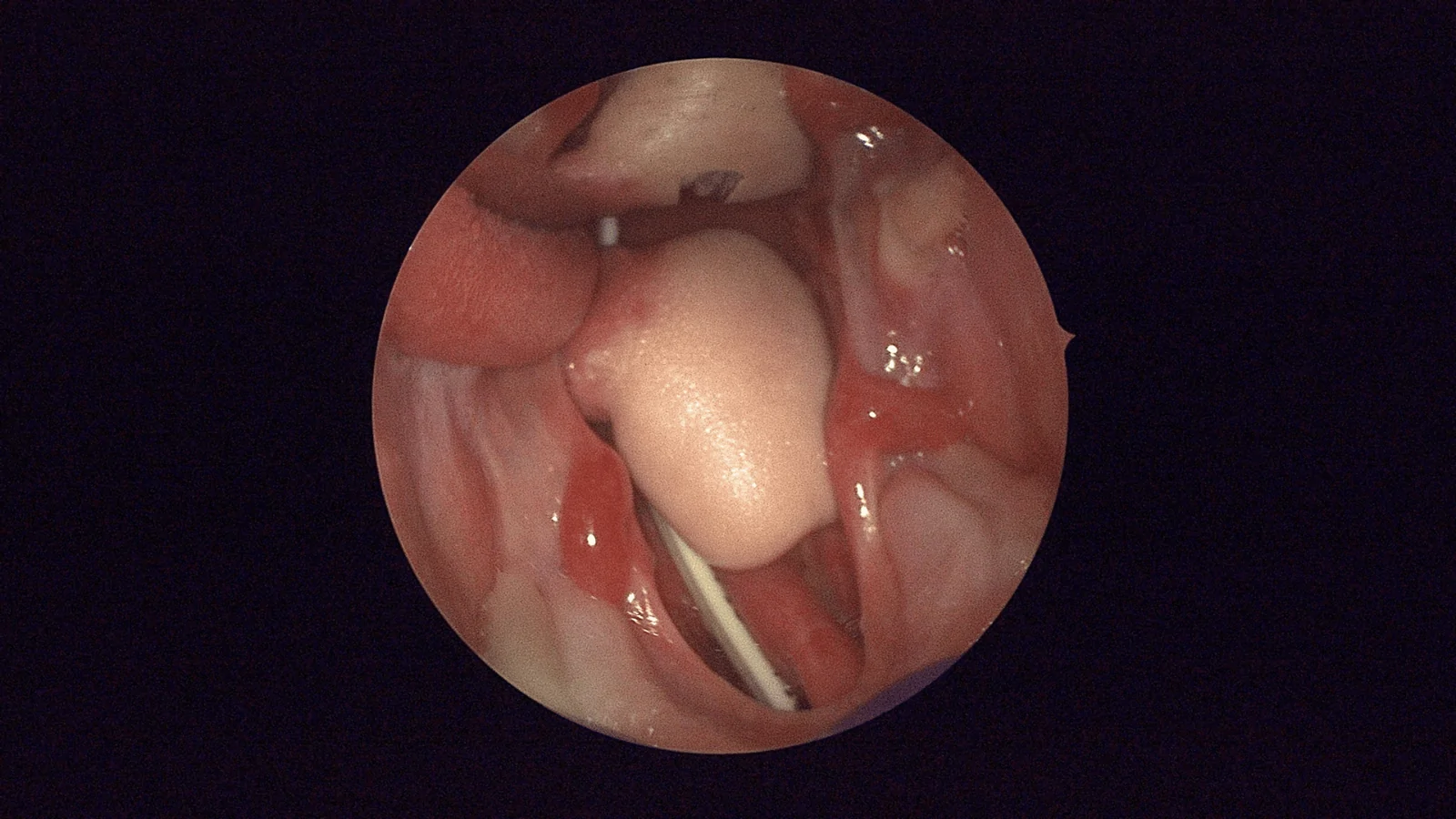
Intraoperative photo of hairy polyp

The child was taken to the theatre on day 3 of birth. The setup was using a similar approach to tonsillectomy using a paediatric Boyle-Davis gag with Draffin bipod rods. Macroscopically, this appeared to be a 3 cm mass attached to the left anterior pillar via a stalk (Figure 3). The lesion was removed at the stalk via coblation (settings 7:3) without any complications (Video 1). A thorough examination of the pharynx and larynx revealed a cleft palate on the ipsilateral side without any laryngeal abnormalities.

**Video 1:** Surgical removal of hairy polyp

The child made an uneventful recovery and was extubated the following day. Histology confirmed a 34 x 22 x 16 mm lesion consisting of keratinized stratified squamous epithelium with pilosebaceous units and a well-formed tooth bell seen within the specimen. This contained both ectodermal and mesodermal elements consistent with a hairy polyp. To this date, the child continues to thrive with no evidence of recurrence or complications.

## Discussion

First described in 1918 by Kelly, hairy polyps are rare benign tumours affecting children of various ages [8,9]. They are described as choristomas, which are tumour-like masses of normal cells presenting at an ectopic location [5]. This is in contrast to hamartomas, where such tumours occur in the mother organ system, i.e., in areas typical of that anatomical location [5,7]. Complex germ-layer lesions have typically been categorized into four entities using Arnold’s classification [2,3,7]: dermoids, e.g., hairy polyps that are composed of two germinal layers, ectoderm and mesoderm; teratoids; teratomas; and epignathi, which are composed of all three germ layers (ectoderm, mesoderm and endoderm). They are distinguished by increasing degrees of differentiation, with teratoids being poorly differentiated and epignathi being the highest form of differentiation in which foetal organs have developed [2,3,7].

Over the years, there have been multiple theories proposed to explain the development of hairy polyps. They were initially thought to result from the escape of pluripotent tissue before the fourth week of gestation. These tissue remnants would become marginated from the standard migratory pathways and give rise to ectopic tissue in various locations [2,7,9,10]. More recently, hairy polyps are thought to arise from developmental abnormalities of the first or second branchial cleft, and this is how they are currently classified in the World Health Organization’s (WHO) latest classification of head and neck tumours [11,12].

Hairy polyps present with an estimated incidence between 1:35000 and 1:200,000, with a female predominance [7,13]. This was initially overestimated in the literature, but more conservative estimates suggest that the ratio of female to male patients is now between 3 and 3.5:1 [7,13]. Interestingly, as in our case, these lesions typically arise from the left side of the body, this side being 6.5 times more common [7]. The reasons for this are yet unknown, but theories include the interplay between sonic hedgehog (shh) gene products and Hox, which influence left-right asymmetry during morphogenesis [5,7,14].

In a review that looked at the most common sites of presentation of hairy polyps, the lateral nasopharyngeal wall was the highest at 29.5%, followed by the Eustachian tube at 19.2%. The least common sites included the lower lip and both the soft and hard palate [7].

Clinical presentation varies, with some cases presenting with airway compromise as in our case. However, a considerable number of patients may remain asymptomatic or present with less severe symptoms at a later age, such as recurrent cough, rhinorrhoea, haemoptysis, feeding difficulties and dysphagia [2,15]. These cases require a high index of suspicion from the clinician assessing the patients [7]. Our patient was found to have a lesion greater than 3 cm in size, which may explain their presentation. Koike et al. have reported that based on a systematic review of 40 hairy polyps, those greater than 3 cm in size were more likely to present with respiratory distress and cardiac arrest and therefore picked up sooner during assessment [16].

Our patient was found to have a cleft palate. Approximately 10% of patients in the literature with hairy polyps have been found to have a cleft palate [11,17]. Although hairy polyps may not be associated with particular congenital syndromes, they are thought to arise due to developmental anomalies of the branchial clefts and may be associated with other defects such as agenesis of the external auricle, absence of the uvula, osteopetrosis, facial hypoplasia and left carotid artery atresia [5,9,11,17,18].

Management of these lesions is usually undertaken through surgical excision. In severe cases, a tracheostomy may need to be performed, especially in cases where prenatal investigations have picked up the mass, in which case an ex-utero intrapartum treatment (EXIT) procedure is planned in advance [13]. Finally, a range of surgical techniques and options are available. We chose to use a coblation device to avoid the increased risk of thermal injury compared to traditional bipolar cautery.

## Conclusions

Hairy polyps are a rare cause of airway compromise in the neonate. We present the case of a newborn who presented in respiratory distress with a larger than 3 cm hairy polyp arising from the oropharynx. Following emergent intubation, this was successfully removed using coblation. The child made an uneventful recovery and has had complete resolution of their symptoms.
